# Targeting CDK2 and AURKA with Cerevisterol from *Ganoderma lucidum* to Sensitize Colorectal Cancer to Chemotherapy

**DOI:** 10.3390/ijms27146120

**Published:** 2026-07-08

**Authors:** Yi Pan, Xuewei Wu, Lin Chen, Chao Zhang, Yuqing Hu, Jie Chang, Qiuwen Lou, Jiaqi Zhang, Shuochen Xu, Wenxia Xu, Jianping Wang

**Affiliations:** 1 Central Laboratory, Precision Medicine Center, Affiliated Jinhua Hospital, Zhejiang University School of Medicine, Jinhua 321000, China; panyi_1995@126.com (Y.P.); xueweiwu_zju@163.com (X.W.); lynne_1121@163.com (L.C.); chaozhang2023@163.com (C.Z.); yqhu721@163.com (Y.H.); jiechang681646@163.com (J.C.); louqiuwen0818@163.com (Q.L.); zjq15988552813@163.com (J.Z.); 15858913428@163.com (S.X.); 2 Jinhua Key Laboratory of Cancer Nutrition and Metabolism Research, Affiliated Jinhua Hospital, Zhejiang University School of Medicine, Jinhua 321000, China; 3 Department of Colorectal Surgery, National Clinical Key Disciplines, Affiliated Jinhua Hospital, Zhejiang University School of Medicine, Jinhua 321000, China

**Keywords:** *Ganoderma lucidum*, colorectal cancer, network pharmacology, single-cell RNA sequencing, cerevisterol, CDK2 and AURKA

## Abstract

Chemotherapy resistance remains a major challenge in colorectal cancer (CRC) treatment, necessitating novel adjuvant strategies. This study employed an integrated analytical strategy combining network pharmacology, single-cell RNA sequencing (scRNA-seq) of patient-derived organoids (PDOs) and molecular dynamics simulations to identify bioactive compounds from *Ganoderma lucidum* and elucidate their chemo-sensitizing mechanisms. Network pharmacology identified five bioactive components of *G. lucidum*, corresponding to 267 potential targets. Integration with transcriptomic data, weighted gene co-expression network analysis (WGCNA), and known CRC genes refined these to 19 core targets. Cross-referencing with scRNA-seq data from irinotecan-treated PDOs pinpointed cyclin-dependent kinase 2 (*CDK2*) and Aurora kinase A (*AURKA*) as pivotal targets. Molecular dynamics simulations confirmed stable binding of the key component cerevisterol to both CDK2 and AURKA proteins, with binding free energies of −120.67 kJ/mol and −134.47 kJ/mol, respectively. In vitro cell viability assays across multiple CRC cell lines (HCT116, RKO, and HT-29) and PDOs demonstrated that cerevisterol significantly sensitized CRC cells to irinotecan (SN38). Notably, we observed that CDK2 was preferentially enriched in MSI-H tumors, whereas AURKA was enriched in MSS tumors, suggesting the potential of MSI status as a biomarker for patient stratification. Collectively, these findings identify cerevisterol as a dual-targeting natural product that modulates CDK2 and AURKA to overcome chemotherapy resistance, providing a quantitative analytical framework for discovering bioactive compounds and their molecular targets from medicinal fungi.

## 1. Introduction

Colorectal cancer (CRC) remains a leading malignancy of the digestive system worldwide, with incidence ranking third and mortality second among all malignant tumors [1]. Current therapeutic strategies, including surgery, chemotherapy, targeted therapy, and immunotherapy, face significant limitations, with chemotherapy resistance representing a major cause of treatment failure [2,3]. This resistance arises from complex mechanisms involving the immunosuppressive tumor microenvironment, ABC transporter-mediated drug efflux, and tumor evolution under therapeutic pressure [4,5,6], leading to reduced efficacy of both conventional agents like 5-FU and targeted therapies such as anti-EGFR antibodies [7,8,9,10]. Overcoming this challenge necessitates novel therapeutic strategies, with natural products offering promising avenues for developing adjunctive therapies [4].

Natural products derived from medicinal fungi and plants have attracted considerable interest as sources of novel adjuvant therapeutic agents [11,12]. Our prior research identified Atractylenolide I as a bioactive compound that exerts anti-CRC effects by activating the DNA damage–apoptosis axis in patient-derived organoids [13]. *Ganoderma lucidum* (*G. lucidum*), a well-known medicinal mushroom, has demonstrated significant pharmacological activities [14,15], but its multi-component profile and the molecular targets responsible for chemotherapy sensitization remain to be systematically characterized using integrated analytical approaches [1,11,12].

The antitumor potential of *G. lucidum* stems from its abundant bioactive components—primarily triterpenoids (e.g., ganoderic acids) and polysaccharides—which have demonstrated broad pharmacological activities. Its extracts have shown inhibitory effects against various solid tumors by inducing apoptosis, suppressing proliferation, and modulating immune responses [1,11]. In CRC, specific triterpenoids can promote apoptosis by modulating Bcl-2 and Bax proteins, while polysaccharides may improve the intestinal microecology by regulating gut microbiota and increasing short-chain fatty acids [1]. Despite these advances, significant research gaps remain. Most studies focus on isolated components or endpoint phenotypes, lacking systematic analysis of multi-target synergistic mechanisms, particularly in the clinically critical context of chemotherapy resistance.

Network pharmacology and multi-omics approaches have emerged as prominent strategies for elucidating the complex mechanisms of natural products. For instance, studies integrating network pharmacology with WGCNA and machine learning have predicted that ginger targets the PRMT1/BTG2 axis in CRC [16]. Other analyses have identified numerous bioactive compounds and targets from medicinal plants against CRC, including *G. lucidum* [17], revealed key targets such as PDGFRA and MAPK1 for andrographolide [18], and highlighted the PI3K-Akt pathway as central to the action of *Ceiba pentandra* [19]. Despite these advances, the specific mechanisms by which *G. lucidum* sensitizes CRC to chemotherapy remain insufficiently explored, highlighting a clear gap for focused investigation.

Based on the above, we hypothesized that *G. lucidum*, through its multi-target nature, could modulate key pathways involved in CRC chemoresistance. We aimed to identify the bioactive compound(s) and their direct targets, and to elucidate the underlying mechanism by which they sensitize CRC to irinotecan. In this study, we integrated network pharmacology with scRNA-seq of PDOs to explore the chemo-sensitizing potential of *G. lucidum* in CRC. Our analyses identified cell cycle regulation as a common mechanism linking chemotherapy resistance and the action of *G. lucidum* in CRC. Targeting the core genes *CDK2* and *AURKA* with the active component cerevisterol sensitized CRC cells to chemotherapy, providing a scientific foundation for translating this medicinal fungus into a modern adjuvant antitumor agent.

## 2. Results

### 2.1. Network Pharmacology Identifies Potential Anti-CRC Targets of G. lucidum

**Figure 1:**
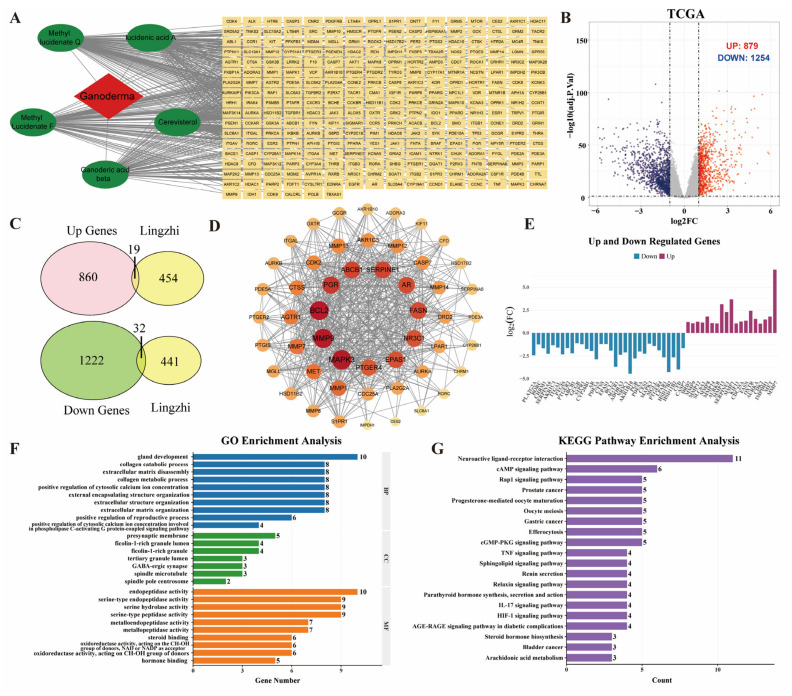
Targets of Ganoderma active ingredients and differentially expressed genes in colorectal cancer using RStudio (R 4.3.1) and Cytoscape 3.10.2. (**A**) Network diagram of Ganoderma active ingredient–target interactions drawn by Cytoscape. (**B**) Volcano plot of differentially expressed genes from the TCGA-COADREAD cohort. (**C**) Intersection of up- and downregulated genes of colorectal cancer and targets of Ganoderma. (**D**) The protein–protein interaction network of 51 core genes. (**E**) LogFC values of the up- and downregulated genes in the intersection targets. (**F**) GO functional enrichment analysis of intersection. (**G**) KEGG pathway enrichment analysis.

To systematically investigate the multi-target mechanisms of *G. lucidum* in CRC, we first employed a network pharmacology approach to screen its bioactive components and corresponding targets. Based on integrated criteria including oral bioavailability (OB ≥ 30%), drug-likeness (DL ≥ 0.10), and gastrointestinal absorption from SwissADME analysis, five bioactive components were identified from *G. lucidum*: cerevisterol, lucidenic acid A, methyl lucidenate Q, ganoderic acid β, and methyl lucidenate F. Their structural information is provided in Table 1 and Table S1, Figure S1. To identify potential targets of these components, we retrieved data from the TCMSP database and supplemented them with target prediction using PubChem and SwissTargetPrediction, yielding a total of 267 candidate targets (Figure 1A; Table S2). Concurrently, to identify CRC-relevant genes, we analyzed transcriptomic data from The Cancer Genome Atlas (TCGA) and identified 2133 genes differentially expressed between tumor and normal tissues (|log_2_FC| > 1, adjusted *p* < 0.05; Figure 1B; Table S3).

To pinpoint potential anti-CRC targets of *G. lucidum*, we performed intersection analysis between the 267 component-derived targets and the 2133 CRC-associated differentially expressed genes. This revealed 51 overlapping genes, including 19 upregulated and 32 downregulated targets in CRC tissues (Figure 1C). A protein–protein interaction (PPI) network was constructed for these 51 genes to explore their functional interconnections (Figure 1D), and their expression fold changes are visualized in Figure 1E. To gain insight into the biological functions of these 51 candidate targets, we performed Gene Ontology (GO) and Kyoto Encyclopedia of Genes and Genomes (KEGG) pathway enrichment analyses. GO analysis revealed significant enrichment in biological processes including gland development, collagen metabolic process, and extracellular matrix organization, as well as molecular functions such as oxidoreductase activity, metallopeptidase activity, and serine-type peptidase activity (Figure 1F). KEGG pathway analysis further highlighted their involvement in key cancer-related signaling pathways, including the p53, cAMP, and TNF signaling pathways (Figure 1G).

Collectively, these initial findings establish the pharmacological profile of *G. lucidum* and identify 51 potential targets that may contribute to its anti-CRC effects, with enrichment in pathways closely linked to tumor progression and inflammation.

### 2.2. WGCNA and Multi-Source Data Integration Prioritize Core Anti-CRC Targets of G. lucidum

**Figure 2:**
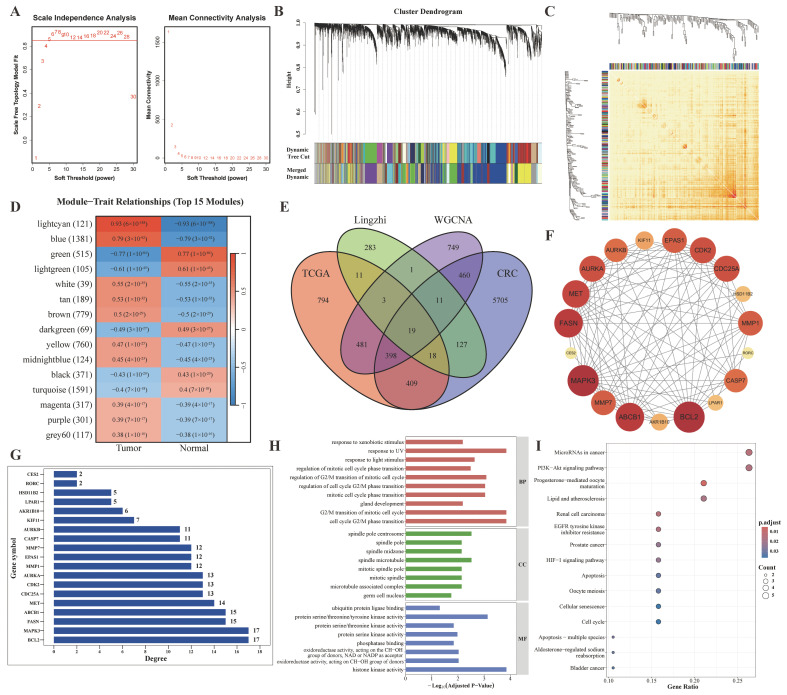
Screening of core genes of *Ganoderma lucidum* for anti-colorectal cancer based on WGCNA and multi-source data integration, with visualization performed using RStudio. (**A**) Screening of soft threshold power in WGCNA. (**B**) Gene co-expression network clustering tree constructed based on selected thresholds. (**C**) Heat map of inter-module correlation. (**D**) Module–trait association analysis, in which red indicates positive correlation and blue indicates negative correlation. (**E**) Four-set cross-Venn diagrams of WGCNA candidate genes, *Ganoderma lucidum* targets, TCGA differentially expressed genes, and known colorectal cancer-related genes. (**F**) The protein–protein interaction network of 19 core genes and (**G**) the distribution of their node degree values. (**H**) GO functional enrichment analysis of core genes. (**I**) KEGG pathway enrichment analysis.

To further narrow down the potential core targets of *G. lucidum* in CRC treatment, we performed weighted gene co-expression network analysis (WGCNA) using TCGA CRC transcriptomic data. Soft-threshold power screening indicated that a threshold of β = 5 optimally satisfied the scale-free topology criterion while maintaining high mean connectivity (Figure 2A). Using this parameter, a co-expression network was constructed, and genes were clustered into 33 distinct modules based on their expression patterns (Figure 2B). A clustering heatmap was generated to visualize correlations among modules, and highly similar modules (merge cut height < 0.25) were subsequently merged (Figure 2C). To identify modules most relevant to CRC pathogenesis, we performed Pearson correlation analysis between module eigengenes and clinically relevant traits. This analysis revealed that the light cyan, light green, green, and blue modules exhibited the strongest correlations with target traits (Figure 2D). After merging highly correlated modules and removing duplicates, a total of 2122 genes were retained as WGCNA-derived candidate genes for subsequent analysis (Table S4).

To pinpoint the most promising anti-CRC targets of *G. lucidum*, we integrated multiple lines of evidence by intersecting four gene sets: (1) the 2122 WGCNA-derived candidate genes, (2) the 267 *G. lucidum* component targets, (3) the 2133 TCGA-derived differentially expressed genes, and (4) 7147 known CRC-related genes from public databases. This multi-dimensional integration yielded 19 overlapping genes (Figure 2E), representing core targets with the highest confidence for mediating the anti-CRC effects of *G. lucidum*.

To explore the functional interrelationships among these 19 core targets, we constructed a protein–protein interaction (PPI) network and calculated node degree values to identify hub genes within the network (Figure 2F,G). Differential expression and survival analyses for each of the 19 core genes are presented in Figure S2. To gain insight into the biological functions and pathways associated with these core targets, we performed GO and KEGG enrichment analyses. GO analysis revealed significant enrichment in biological processes related to mitotic cell cycle, serine metabolism, and oxidoreductase activity (Figure 2H). KEGG pathway analysis demonstrated that these core targets are primarily involved in cell cycle-related pathways, including the PI3K-Akt signaling pathway, apoptosis, and cell cycle regulation (Figure 2I).

Collectively, these findings indicate that the 19 core targets of *G. lucidum* are predominantly enriched in cell cycle-related biological processes and pathways, suggesting that *G. lucidum* may exert its anti-CRC effects primarily through modulation of cell cycle progression.

### 2.3. Single-Cell Transcriptomic Characterization and Functional Analysis of Chemotherapy-Sensitive and Resistant CRC Organoids

**Figure 3:**
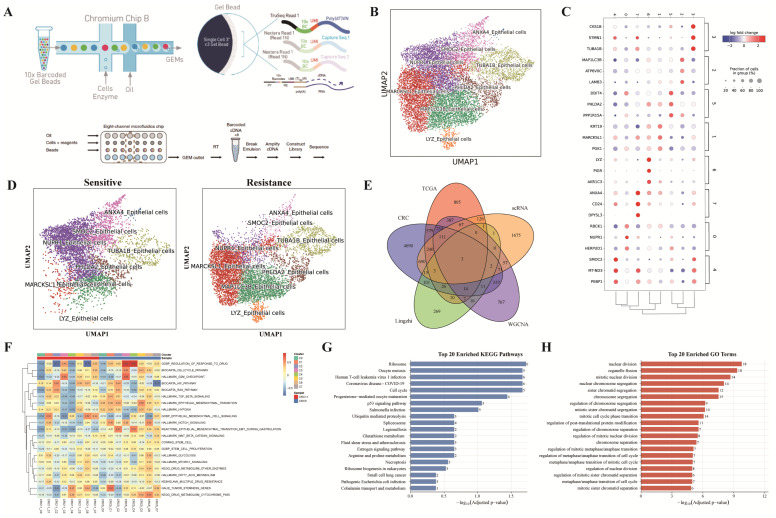
Single-cell transcriptome characteristics and functional analysis of chemotherapy-sensitive and drug-resistant colorectal cancer organoids using RStudio. (**A**) Schematic diagram illustrating the single-cell RNA sequencing workflow (cited from the 10× Genomics official website). (**B**,**C**) UMAP dimensionality reduction and clustering visualization of the scRNA-seq data from organoids. (**D**) The expression and distribution of the top three marker genes in each identified cell subpopulation. (**E**) Venn diagram showing the intersection between single-cell differentially expressed genes and core genes identified from network pharmacology. (**F**) Heat map from gene set variation analysis (GSVA), depicting the differences in the activity of key signaling pathways between sensitive and resistant organoids. (**G**) GO functional enrichment analysis of the differentially expressed genes in Cluster 3. (**H**) KEGG pathway enrichment analysis of the differentially expressed genes in Cluster 3.

To determine which of the 19 core targets might be functionally involved in chemotherapy response, we re-analyzed our recently published scRNA-seq data from CRC PDOs classified as either irinotecan-sensitive or irinotecan-resistant [20] (Figure 3A). Comparative analysis revealed distinct shifts in cellular heterogeneity and marker gene expression between sensitive and resistant organoids (Figure 3B–D), suggesting that chemotherapy resistance is associated with transcriptional remodeling at single-cell resolution. To identify core targets potentially driving this resistance phenotype, we intersected the 19 core genes with the differentially expressed genes identified from the scRNA-seq dataset. This analysis yielded three overlapping genes: *AURKA*, *CDK2*, and kinesin family member 11 (*KIF11)* (Figure 3E). Notably, all three genes were specifically enriched in the same cell subpopulation—Cluster 3, annotated as TUBA1B^+^ epithelial cells (Table S5)—suggesting that this subpopulation may harbor a resistance-associated transcriptional program.

To explore the functional significance of Cluster 3 in chemotherapy resistance, we performed gene set variation analysis (GSVA) comparing sensitive versus resistant organoids within this subpopulation. The results revealed significant differences in the activity of multiple pathways, with cell cycle-related pathways showing the most pronounced enrichment in resistant organoids (Figure 3F). This observation was further supported by GO and KEGG enrichment analyses of the differentially expressed genes in Cluster 3, which identified chromosome segregation, cell cycle regulation, and the p53 signaling pathway as the most significantly enriched terms (Figure 3G,H).

Collectively, these single-cell transcriptomic findings pinpoint *AURKA* and *CDK2* (along with *KIF11*) as core targets enriched in a distinct epithelial subpopulation, where they are functionally associated with dysregulated cell cycle progression and DNA damage response—key hallmarks of chemotherapy resistance in CRC.

### 2.4. Pan-Cancer Expression and Immune Landscape of the Candidate Targets

Among the three core genes identified from single-cell analysis, *KIF11* was excluded from subsequent investigation due to its lack of significant association with overall survival in CRC patients (Figure S2N). In contrast, both *CDK2* and *AURKA* demonstrated significant clinical relevance and were therefore selected for further pan-cancer characterization to contextualize their roles in CRC within a broader oncogenic landscape.

**Figure 4:**
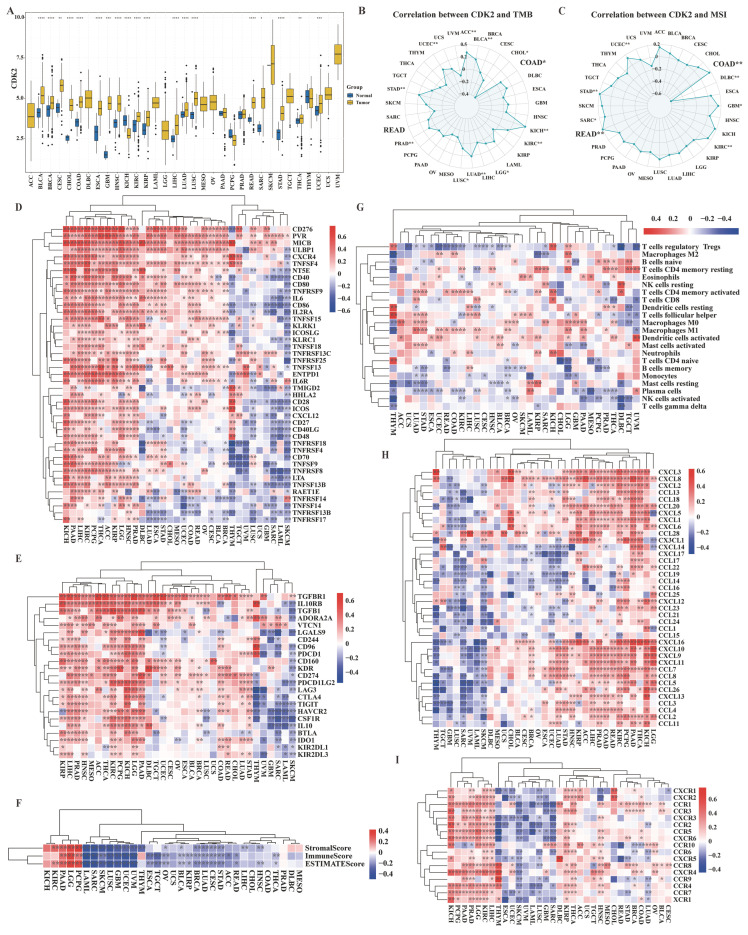
Pan-cancer landscape and immunological correlations of *CDK2* using RStudio (* *p* < 0.05, ** *p* < 0.01, *** *p* < 0.001, **** *p* < 0.0001). (**A**) Differential expression of *CDK2* between tumor and normal tissues across multiple cancer types; (**B**–**I**) Correlation between *CDK2* expression and (**B**) TMB; (**C**) MSI; (**D**) immunostimulatory molecules expression; (**E**) immunosuppressive molecules expression; (**F**) immune score; (**G**) immune cell infiltration levels; (**H**) chemokine expression; (**I**) chemokine receptor expression in pan-cancer.

**Figure 5:**
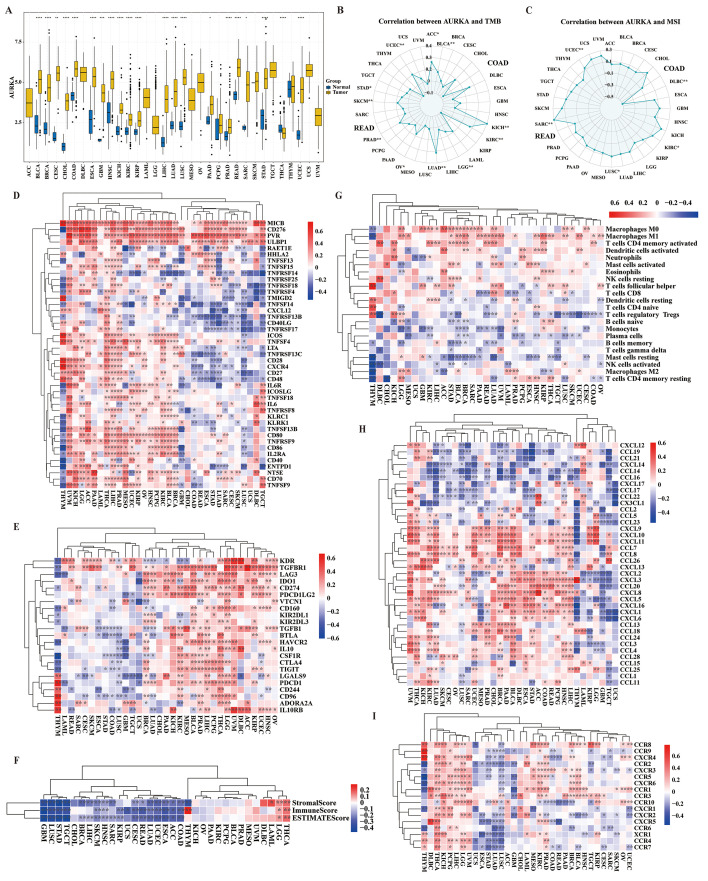
Pan-cancer landscape and immunological correlations of *AURKA* using RStudio (* *p* < 0.05, ** *p* < 0.01, *** *p* < 0.001, **** *p* < 0.0001). (**A**) Differential expression of *AURKA* between tumor and normal tissues across multiple cancer types. (**B**–**I**) Correlation between *AURKA* expression and (**B**) TMB; (**C**) MSI; (**D**) immunostimulatory molecules expression; (**E**) immunosuppressive molecules expression; (**F**) immune score; (**G**) immune cell infiltration levels; (**H**) chemokine expression; (**I**) chemokine receptor expression in pan-cancer.

Using TCGA multi-cancer datasets, we first evaluated the differential expression of *CDK2* and *AURKA* across tumor types. *CDK2* expression was significantly elevated in tumor tissues compared to normal counterparts in 18 cancer types (Figure 4A), while *AURKA* was upregulated in 20 cancer types (Figure 5A), indicating their widespread involvement in oncogenesis. We next examined their associations with key genomic features of tumor aggressiveness. *CDK2* expression correlated significantly with tumor mutational burden (TMB) in 12 cancers and with microsatellite instability (MSI) in 8 cancers (Figure 4B,C). Consistently, *AURKA* expression showed significant correlations with TMB in 11 cancers and with MSI in 5 cancers (Figure 5B,C), further supporting their roles as broad drivers of tumor progression. Given the established interplay between cell cycle regulators and the tumor immune microenvironment, we systematically investigated the immunological correlates of *CDK2* and *AURKA* across cancer types. Both genes exhibited widespread correlations with immunomodulatory molecules, including immunostimulatory and immunosuppressive factors, as well as with immune scores, immune cell infiltration levels, chemokines, and chemokine receptors (Figure 4D–I and Figure 5D–I). These findings suggest that *CDK2* and *AURKA* may not only regulate cell cycle progression but also modulate immune responses in the tumor microenvironment, with potential implications for immunotherapy response.

Collectively, this pan-cancer analysis establishes *CDK2* and *AURKA* as broadly upregulated oncogenic drivers with significant immunological relevance, providing a strong rationale for their focused investigation in CRC.

### 2.5. Clinical Relevance and Experimental Validation of CDK2 and AURKA in CRC

**Figure 6:**
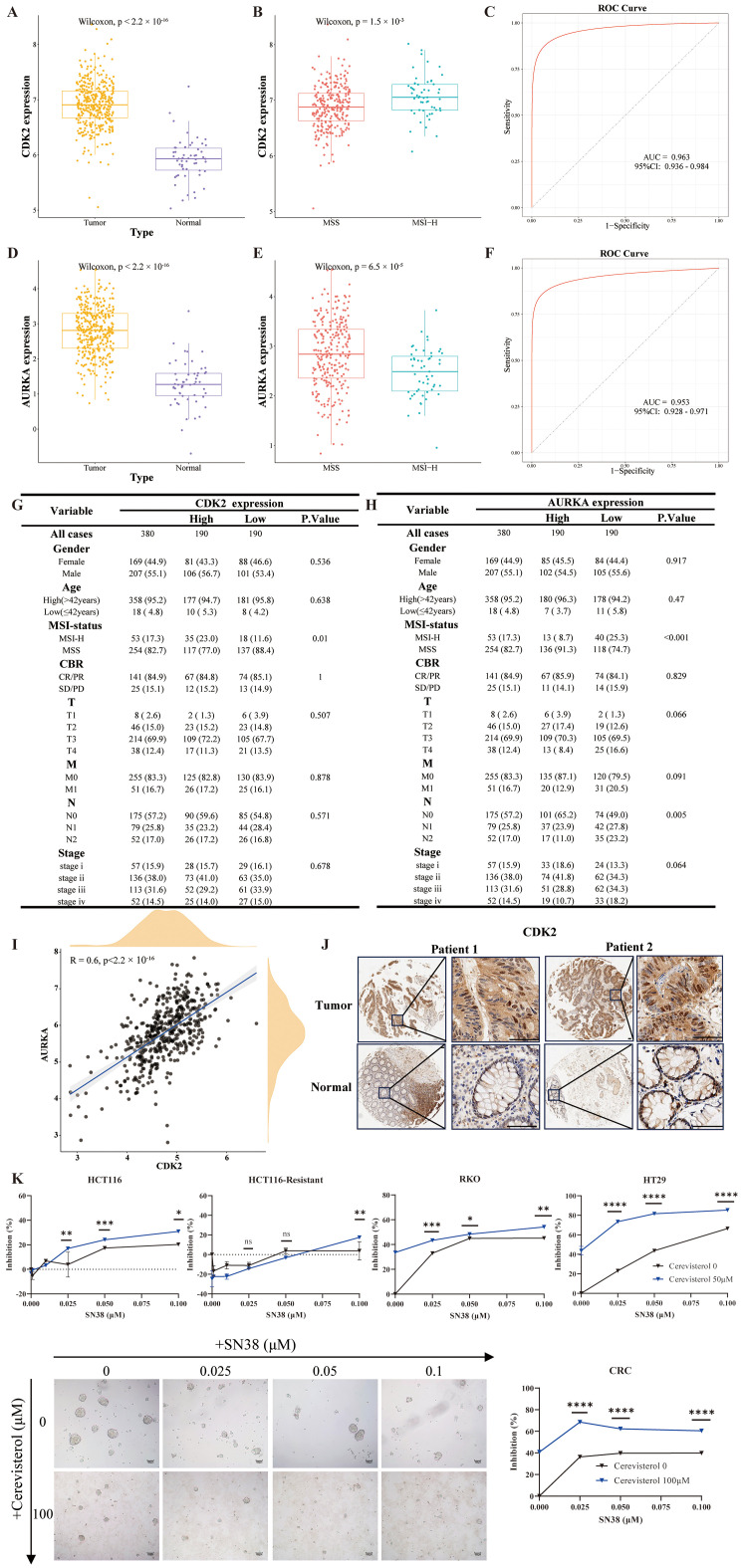
Clinical relevance and experimental validation of *CDK2* and *AURKA* in colorectal cancer (* *p* < 0.05, ** *p* < 0.01, *** *p* < 0.001, **** *p* < 0.0001). (**A**) Differential expression of *CDK2* between tumor and normal tissues in the TCGA cohort. (**B**) Comparison of *CDK2* expression between MSI-H and MSS subtypes. (**C**) ROC curve of *CDK2* for predicting colorectal cancer. (**D**) Differential expression of *AURKA* between tumor and normal tissues in the TCGA cohort. (**E**) Comparison of *AURKA* expression between MSI-H and MSS subtypes. (**F**) ROC curve of *AURKA* for predicting colorectal cancer. (**G**,**H**) Clinical baseline characteristics of patients stratified by (**G**) *CDK2* and (**H**) *AURKA* expression in the TCGA cohort. (**I**) Correlation analysis between *CDK2* and *AURKA* expression. (**J**) IHC staining of CDK2 protein in patient tissues. (**K**) Cell viability assay following combination treatment with cerevisterol and SN38 (graph generated using GraphPad Prism 9.5.0). The scale bar in the microscopic images represents 100 μm.

To further validate the clinical significance of *CDK2* and *AURKA* in CRC, we analyzed their expression patterns and clinical correlations using the TCGA-COADREAD cohort. Consistent with our earlier findings, both *CDK2* (Figure 6A) and *AURKA* (Figure 6D) were significantly upregulated in tumor tissues compared to normal counterparts. Notably, when stratifying patients by microsatellite instability status, we observed distinct expression patterns: *CDK2* expression was significantly higher in MSI-H patients (Figure 6B), whereas *AURKA* was more highly expressed in MSS patients (Figure 6E). Examination of other clinical parameters revealed no significant associations for either gene, with the exception of a weak negative correlation between *AURKA* expression and lymph node count (Figure S3). Receiver operating characteristic (ROC) curve analysis demonstrated strong diagnostic performance for both genes, with AUC values of 0.963 for *CDK2* and 0.953 for *AURKA* (Figure 6C,F), underscoring their potential as diagnostic biomarkers. Further analysis confirmed that *CDK2* expression varied significantly only by MSI status, whereas *AURKA* expression was associated with both MSI status and lymph node involvement (Figure 6G,H). Correlation analysis revealed a significant positive association between *CDK2* and *AURKA* expression in CRC (R = 0.60, *p* < 2.2×10^−16^; Figure 6I), suggesting potential co-regulation or functional cooperation between these two cell cycle kinases.

To validate these bioinformatic findings at the protein level, we performed IHC on a CRC tissue microarray. Consistent with the transcriptomic data, CDK2 protein exhibited strong positive staining in tumor tissues, while adjacent normal tissues showed weak or negative staining (Figure 6J), confirming its tumor-associated upregulation.

Finally, to functionally assess whether targeting *CDK2* and *AURKA* could enhance chemotherapy efficacy, which was a central hypothesis of this study, we treated HCT116, HCT116-resistant, RKO, HT-29 cells, as well as PDOs, with cerevisterol in combination with SN38. As shown in Figure 6K, cerevisterol treatment significantly reduced cell viability compared to SN38 alone across all models, demonstrating that cerevisterol effectively sensitizes CRC cells to irinotecan-induced cytotoxicity.

Collectively, these results establish the clinical relevance of *CDK2* and *AURKA* in CRC and provide functional evidence that cerevisterol, through targeting these cell cycle regulators, enhances chemotherapy sensitivity.

### 2.6. Molecular Docking and Dynamics Simulations Confirm Stable Binding of Cerevisterol to CDK2 and AURKA Proteins

**Figure 7:**
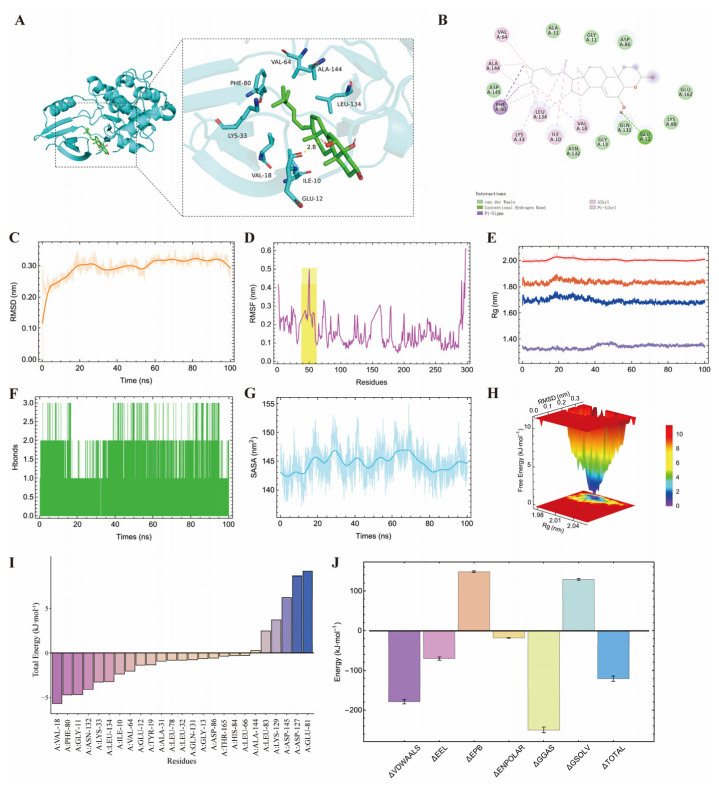
Computational analysis of cerevisterol binding to CDK2 proteins using PyMOL, Discovery Studio 2019, and GROMACS. (**A**) Predicted three-dimensional binding pose of cerevisterol (green) within CDK2 (cyan). (**B**) Two-dimensional schematic of key molecular interactions. (**C**–**J**) Molecular dynamics simulation trajectories assessing complex stability: (**C**) RMSD; (**D**) RMSF; (**E**) Rg; (**F**) number of hydrogen bonds; (**G**) SASA; (**H**) Gibbs free energy landscape; (**I**) Residue-wise energy contributions of CDK2 interacting with cerevisterol; (**J**) Decomposed total binding free energy of the CDK2-cerevisterol complex.

**Figure 8:**
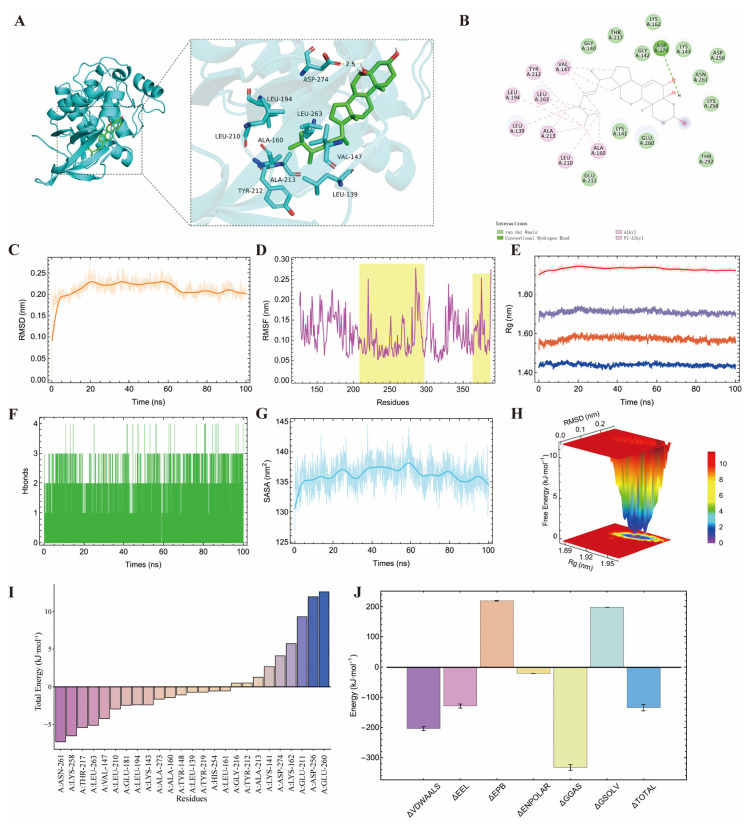
Computational analysis of Cerevisterol binding to AURKA proteins using PyMOL, Discovery Studio 2019, and GROMACS. (**A**) Predicted three-dimensional binding pose of cerevisterol (green) within AURKA (cyan). (**B**) Two-dimensional schematic of key molecular interactions. (**C**–**J**) Molecular dynamics simulation trajectories assessing complex stability: (**C**), RMSD; (**D**) RMSF; (**E**) Rg; (**F**) number of hydrogen bonds; (**G**) SASA; (**H**) Gibbs free energy landscape; (**I**) Residue-wise energy contributions of AURKA interacting with cerevisterol; (**J**) Decomposed total binding free energy of the AURKA-cerevisterol complex.

To experimentally validate whether cerevisterol, the key bioactive component identified from *G. lucidum*, could directly interact with CDK2 and AURKA, we performed molecular docking followed by 100 ns molecular dynamics (MD) simulations. Molecular docking revealed favorable binding affinities of cerevisterol to both targets, with docking scores of −9.2 kcal/mol for CDK2 and −8.6 kcal/mol for AURKA (Figure 7A,B and Figure 8A,B). Analysis of the binding modes indicated that the complexes were primarily stabilized by hydrogen bonding and hydrophobic interactions. Specifically, cerevisterol formed a hydrogen bond with GLU12 (2.8 Å) in CDK2, while hydrophobic contacts involved residues ILE10, VAL18, LYS33, VAL64, PHE80, LEU134, and ALA144 (Figure 7B). For AURKA, a hydrogen bond was observed with ASP274 (2.5 Å), surrounded by a hydrophobic pocket comprising LEU139, VAL147, ALA160, LEU194, LEU210, TYR212, ALA213, and LEU263 (Figure 8B).

To assess the stability and dynamic behavior of these interactions, we conducted 100 ns MD simulations. The root-mean-square deviation (RMSD) trajectories showed that both systems reached equilibrium after initial stabilization, with average RMSD values of 0.29 nm for CDK2 and 0.20 nm for AURKA (Figure 7C and Figure 8C), indicating conformational stability throughout the simulation. Root-mean-square fluctuation (RMSF) analysis revealed enhanced flexibility in specific regions—residues 40–60 for CDK2 and residues 21–300 and 360–380 for AURKA—suggesting these segments may be involved in ligand accommodation (Figure 7D and Figure 8D). Additional structural parameters further confirmed complex stability. The radius of gyration (Rg) remained stable at approximately 2.01 nm for CDK2 and 1.925 nm for AURKA (Figure 7E and Figure 8E), indicating compact folding. Hydrogen bond analysis showed that 1–3 hydrogen bonds in the CDK2–cerevisterol complex and 1–4 bonds in the AURKA–cerevisterol complex were consistently maintained throughout the simulation (Figure 7F and Figure 8F). Solvent-accessible surface area (SASA) fluctuated within narrow ranges for both complexes (Figure 7G and Figure 8G). Free energy landscapes constructed from RMSD and Rg values displayed a single deep energy well for each system (Figure 7H and Figure 8H), further corroborating their conformational stability.

Per-residue energy decomposition identified key contributors to binding: VAL18, PHE80, and GLY11 for CDK2 (Figure 7I), and ASN261, LYS258, and THR217 for AURKA (Figure 8I). Finally, MM/PBSA binding free energy calculations yielded average values of −120.67 kJ/mol for CDK2 and −134.47 kJ/mol for AURKA (Figure 7J and Figure 8J), providing quantitative evidence for strong and stable binding.

Collectively, these computational analyses demonstrate that cerevisterol binds directly and stably to both CDK2 and AURKA, supporting its role as a dual-targeting bioactive compound from *G. lucidum*.

## 3. Discussion

CRC remains a leading cause of cancer-related morbidity and mortality worldwide, underscoring the urgent need for safer and more effective therapeutic strategies [1]. Natural products, characterized by their multi-target and multi-component features, have emerged as promising sources of adjuvant agents to enhance chemotherapy efficacy while mitigating toxicity [11]. In this study, we employed an integrative strategy combining network pharmacology with scRNA-seq of PDOs to investigate the chemo-sensitizing mechanisms of *G. lucidum* in CRC. Our approach identified cerevisterol, a bioactive component of *G. lucidum*, as a dual-targeting agent that directly binds to the cell cycle regulators CDK2 and AURKA, thereby sensitizing CRC cells to irinotecan.

Previous studies have established that *G. lucidum* and its active constituents—particularly triterpenoids and polysaccharides—exhibit broad antitumor activities in CRC, including inhibition of proliferation, induction of apoptosis, and modulation of the tumor immune microenvironment [12]. However, these investigations have largely focused on isolated components or endpoint phenotypes, lacking a systematic analysis of the multi-target synergistic regulatory networks underlying its therapeutic effects. This gap is particularly evident in the clinically critical context of chemotherapy resistance, which remains poorly understood. To address this limitation, we integrated, for the first time, network pharmacology, WGCNA, and scRNA-seq of PDOs to construct an active component–core target–functional module analytical framework. Our analysis revealed that the potential targets of *G. lucidum* active components are significantly enriched in cell cycle regulation pathways. Furthermore, by intersecting with a CRC chemotherapy resistance gene set derived from PDOs, we pinpointed *CDK2* and *AURKA* as pivotal hub genes linking the resistant phenotype to the action of *G. lucidum*.

The CDK2 and AURKA proteins are both key regulators of cell cycle progression. CDK2 has been extensively implicated in CRC pathogenesis, positioning its inhibition as a promising therapeutic strategy [21]. Synthetic CDK2 inhibitors such as SU9516 and CVT-313 have been shown to induce cell cycle arrest and apoptosis in cancer cells [22,23]. However, the efficacy of single-agent CDK2 inhibition is often limited. For instance, CVT-313 alone exhibited minimal activity in patient-derived CRC cell lines, with its antitumor effects becoming significantly enhanced only when combined with CDK9 inhibition [24]. Similarly, while the selective CDK2 inhibitor INX-315 potently induces cell cycle arrest and senescence, maximal sensitivity in cancer cells frequently requires co-targeting strategies, such as concomitant inhibition of CDK4/6 [25]. *AURKA* expression is positively correlated with CRC progression [26], although its prognostic role appears stage-dependent. While high *AURKA* expression has been associated with favorable outcomes in stage II CRC, its inhibition shows therapeutic benefit in advanced metastatic disease [27]. Additionally, AURKA represents a promising target for ARID1A-deficient CRC [28], and its knockdown has been shown to sensitize CRC cells to radiotherapy [29]. These observations highlight the therapeutic potential of targeting CDK2 and AURKA, while also underscoring the context-dependent limitations of their direct pharmacological inhibition—a challenge that may be addressed by alternative targeting strategies, such as the use of multi-target natural compounds. Furthermore, although KIF11 was also found to be enriched in the resistant subpopulation, it was not pursued further, as it primarily participates in spindle assembly rather than cell cycle regulation, lacks prognostic value in CRC, and exhibits relatively weak interaction with cerevisterol.

In our study, we also observed an interesting phenomenon: *CDK2* was enriched in MSI-H tumors, whereas *AURKA* was enriched in MSS tumors. In MSI-H CRC, CDK2 overexpression may result from recurrent frameshift mutations in the *CDK2*-AP1 gene, which impair its negative regulation of CDK2 activity [30]. In MSS tumors, *AURKA* overexpression has been linked to chromosomal instability, a hallmark of this subtype. Beyond cell-cycle control, *AURKA* also critically modulates the tumor immune microenvironment; recent evidence indicates that *AURKA* activation enhances CD4^+^ T-cell infiltration and may predict immunotherapy response in CRC [31]. Collectively, these distinct dependencies suggest that MSI status could serve as a biomarker to stratify patients for CDK2- or AURKA-targeted chemo-sensitization strategies, and we plan to investigate this hypothesis in our future work.

Cerevisterol, a sterol derivative isolated from various medicinal fungi, has previously been reported to exert anti-inflammatory effects by suppressing the MAPK/NF-κB/AP-1 pathway and activating the Nrf2/HO-1 cascade [32]. It has also been shown to interact with epigenetic regulators such as EP300 and STAT3 [33], and to exhibit cytotoxicity against breast cancer cells [34]. In this study, we identified cerevisterol as a bioactive component of *G. lucidum* capable of directly and stably binding to both CDK2 and AURKA, as demonstrated by molecular docking and 100 ns molecular dynamics simulations. Notably, rather than exerting direct cytotoxicity against CRC cells, cerevisterol functioned primarily as a chemo-sensitizing agent, significantly enhancing the sensitivity of multiple CRC cell lines (HCT116, RKO, and HT-29) and PDOs to SN38, the active metabolite of irinotecan. This dual-targeting, chemo-sensitizing mechanism represents a previously unreported mode of action for cerevisterol and provides a molecular rationale for the use of natural products derived from medicinal fungi and plants as sources of novel adjuvant therapeutic agents [11,12].

Despite these promising findings, several limitations of this study should be acknowledged. First, while our target identification and prioritization relied primarily on computational approaches—including network pharmacology, WGCNA, and molecular dynamics simulations—and we have demonstrated stable binding of cerevisterol to CDK2 and AURKA through molecular simulations, these findings warrant further experimental validation through techniques such as surface plasmon resonance or cellular thermal shift assays to definitively confirm direct physical interactions. Second, although our study focused on CDK2 and AURKA as core targets, the multi-target nature of *G. lucidum* suggests that other potential targets may also contribute to its chemo-sensitizing effects. Therefore, a more comprehensive investigation of the broader target landscape of cerevisterol and other bioactive components of *G. lucidum* is warranted to fully elucidate its multi-target synergistic mechanisms. Addressing these limitations in future studies will further strengthen the translational potential of our findings.

## 4. Materials and Methods

### 4.1. Prediction and Acquisition of G. lucidum Targets

The medicinal fungus *Ganoderma lucidum* was used in this study. Its species name was verified against the Index Fungorum database (www.indexfungorum.org, accessed on 7 May 2026), and confirmed as an accepted name. To investigate its bioactive constituents, the Traditional Chinese Medicine Systems Pharmacology Database and Analysis Platform (TCMSP, https://www.tcmsp-e.com/, accessed on 7 May 2026) was employed to retrieve the active ingredients of *G. lucidum*. Preliminary screening was performed using the criteria of oral bioavailability (OB) ≥ 30% and drug-likeness (DL) ≥ 0.10. The SMILES structures of each candidate ingredient were obtained from the PubChem database (https://pubchem.ncbi.nlm.nih.gov/, accessed on 7 May 2026) and subsequently submitted to the SwissADME database (http://www.swissadme.ch/, accessed on 7 May 2026) for further evaluation based on gastrointestinal absorption and compliance with the five rules of drug-likeness. Finally, potential targets for the confirmed active ingredients were predicted using the SwissTargetPrediction database (http://www.swisstargetprediction.ch/, accessed on 7 May 2026).

### 4.2. CRC Target Acquisition and Differential Expression Genes (DEGs) Screening

Targets associated with CRC were retrieved from the GeneCards database (https://www.genecards.org, accessed on 7 May 2026), the OMIM database (https://www.omim.org, accessed on 7 May 2026), and the DisGeNET database (https://www.disgenet.org, accessed on 7 May 2026). Transcriptomic data related to CRC were obtained from The Cancer Genome Atlas (TCGA; https://www.cancer.gov/ccg/research/genome-sequencing/tcga, accessed on 7 May 2026). DEGs between tumor and normal tissues were identified using the limma package (v3.58.1) in R, with significance thresholds set at |log2(fold change)| > 1 and adjusted *p*-value < 0.05.

### 4.3. Weighted Gene Co-Expression Network Analysis (WGCNA) Screening for Module-Associated Targets

A weighted gene co-expression network was constructed using the WGCNA package (v1.74) in R. Low-expressed genes (FPKM < 1 in over 50% of samples) were filtered prior to analysis. The optimal soft-thresholding power (β) was selected based on the scale-free topology fit criterion to ensure a biologically relevant co-expression network. Modules were identified through dynamic tree-cutting with a minimum module size of 30, and highly similar modules (merge cut height < 0.25) were subsequently merged. Module–trait associations were evaluated by correlating module eigengenes with immune and invasive-related phenotypic traits. Hub genes within trait-associated modules were identified based on high intramodular connectivity, with a threshold of kME (module membership) > 0.8.

### 4.4. Construction of Protein–Protein Interaction (PPI) Network and Enrichment Analysis

The screened gene targets were imported into the STRING database to construct a PPI network. CytoScape 3.9.1 software was used to filter core targets and build the PPI network topology. The clusterProfiler package (v4.9.0) in R software was employed to perform Gene Ontology (GO) biological function and Kyoto Encyclopedia of Genes and Genomes (KEGG) pathway enrichment analyses for the core targets, and the results were visualized.

### 4.5. Single-Cell RNA Sequencing Analysis of CRC Patient-Derived Organoids (PDOs)

scRNA-seq data from our previous study, comprising irinotecan-sensitive and irinotecan-resistant CRC PDOs, were re-analyzed. Data processing, normalization, and clustering were conducted using the Seurat package (v4.0.4). Dimensionality reduction and visualization were performed via UMAP. Differential expression analysis for each cell cluster was carried out with the FindAllMarkers function (Wilcoxon rank-sum test, average |log2(fold change)| ≥ 0.25). Gene set variation analysis (GSVA, v1.50.0) was applied to normalized single-cell data using gene sets from the Molecular Signatures Database (MSigDB) to evaluate pathway activity differences between cell subpopulations.

### 4.6. Pan-Cancer Immune Landscape Analysis of Target Genes

To evaluate the pan-cancer immunological relevance of the target genes, transcriptomic profiles and immune-associated data across 33 cancer types were obtained from TCGA. Data were normalized, and batch effects were removed using the sva package (v3.48.0). Subsequent analyses were conducted with the TCGAplot package (v8.0.0) in R. The expression of each target gene was correlated with immune cell infiltration levels, immune checkpoint gene expression, and tumor mutation burden (TMB). Spearman’s correlation coefficient was used to assess the strength of associations, and a *p*-value < 0.05 was considered statistically significant.

### 4.7. Immunohistochemistry (IHC) Analysis of Tumor Samples

CRC tissue samples were obtained from the Department of Colorectal Surgery, Affiliated Jinhua Hospital, Zhejiang University School of Medicine. Tissue microarrays were prepared using an automated tissue microarrayer (AUTO 12A, Nanguang, Guangzhou, China). IHC staining was performed by the Department of Pathology using an anti-CDK2 protein antibody (ET1602-6, HUABIO, Hangzhou, China) and anti-AURKA (ET1609-22, HUABIO, Hangzhou, China) antibody according to standard protocols.

### 4.8. Molecular Docking

The three-dimensional structure of the compound was retrieved from the PubChem database. Molecular docking was performed using AutoDock Vina (v1.2.5), with a genetic algorithm employed for conformational sampling and scoring. The resulting poses were ranked based on docking scores, and the top-ranking conformation was selected for subsequent binding mode analysis. Visualization and graphical representation of the docking results were carried out using PyMOL (version 3.0.3) and Discovery Studio 2019.

### 4.9. Molecular Dynamics Simulation

A 100 ns molecular dynamics (MD) simulation was conducted on the docked complex using GROMACS (v2024.2). The system was modeled with the Amber99sb force field and the TIP3P water model, and neutralized by adding Na^+^ or Cl^−^ ions. Energy minimization was first performed using the steepest descent algorithm, followed by equilibration in the NVT ensemble (100 ps, 300 K) and then in the NPT ensemble (100 ps, 300 K, 1 bar). Subsequently, a production MD run was carried out for 100 ns for further analysis. From the resulting trajectory, the root-mean-square deviation (RMSD), root-mean-square fluctuation (RMSF), radius of gyration (Rg), solvent-accessible surface area (SASA), and the number of hydrogen bonds were calculated. A three-dimensional Gibbs free energy landscape was generated based on RMSD and Rg values. Furthermore, the binding free energy of the complex was quantitatively estimated using the MM-PBSA method implemented in the gmx_MMPBSA module (v1.6.3). Energy decomposition analysis was also performed to evaluate the contribution of key residues to the binding interaction.

### 4.10. Cell Treatment and Viability Assay

Cerevisterol (CAS 516-37-0, purity 99.88%, Cat. No. HY-N3571) was purchased from MedChemExpress (Monmouth Junction, NJ, USA). Human colorectal cancer cell lines HCT116 (NCBI BioSample: SAMN10482761), RKO (SAMN06044803), and HT-29 (SAMN50605044) were obtained from the Shanghai Institute of Cell Research, Chinese Academy of Sciences (Shanghai, China). All cell lines were maintained at 37 °C in a 5% CO_2_ atmosphere. To establish drug-resistant cell models, cells were exposed to gradually increasing concentrations of SN38 over a period of approximately 6 months, starting from 0 and progressively increasing to the IC_50_ level. For viability assays, cells were seeded, cultured for 24 h, and then treated with the indicated concentrations of cerevisterol and SN38 for 72 h. Cell viability was determined using the CCK-8 assay kit (Beyotime, Shanghai, China, Cat. # C0041). Briefly, CCK-8 reagent was added to each well and incubated at 37 °C for 1 h, after which the absorbance was measured at 450 nm.

PDOs were cultured as previously described [20]. For drug treatment, PDOs were cultured for 3 days and then exposed to the indicated concentrations of cerevisterol and SN38 for 72 h. Organoid morphology was documented under a microscope at the end of the treatment period. Cell viability was assessed using the CCK-8 assay kit (Beyotime, Cat. # C0042), with absorbance read at 450 nm following a 1 h incubation with the reagent.

### 4.11. Statistical Analysis

All statistical analyses were performed using R (version 4.3.1). Normality and homogeneity of variance were assessed using the Shapiro–Wilk test and Levene’s test, respectively. Data meeting normality and homogeneity of variance assumptions were analyzed using parametric tests (independent t-test for two groups; ANOVA for multiple groups). Otherwise, non-parametric tests were applied (Wilcoxon rank-sum test for two groups; Kruskal–Wallis H-test for multiple groups). Correlation analyses were performed using Pearson’s or Spearman’s method. A *p*-value < 0.05 was considered statistically significant.

## 5. Conclusions

This study established an integrated strategy combining network pharmacology, scRNA-seq of PDOs, and molecular dynamics simulations to identify bioactive components from *Ganoderma lucidum* and elucidate their chemo-sensitizing mechanisms in CRC. Five bioactive components were identified, with cerevisterol as the lead compound. Target prioritization revealed CDK2 and AURKA as core targets, and molecular dynamics simulations confirmed stable binding of cerevisterol to both targets, with binding free energies of −120.67 kJ/mol and −134.47 kJ/mol, respectively. Notably, we observed that CDK2 was preferentially enriched in MSI-H tumors, whereas AURKA was enriched in MSS tumors, suggesting that MSI status may serve as a potential biomarker for patient stratification in future chemo-sensitization strategies. In vitro experiments across multiple CRC cell lines (HCT116, RKO, HT-29) and PDOs consistently demonstrated that cerevisterol significantly enhanced the sensitivity of CRC cells to SN38. Collectively, these findings identify cerevisterol as a dual-targeting natural product that modulates CDK2 and AURKA to overcome chemotherapy resistance, providing a scientific foundation for its development as a novel adjuvant agent for CRC chemotherapy.

## Figures and Tables

**Table 1:** The structure of the 5 compounds of *Ganoderma lucidum*.

Molecule Name	CID	OB	DL	Molecular Formula	SMILES
Cerevisterol	10181133	37.96	0.77	C_28_H_46_O_3_	C[C@H](/C=C/[C@H](C)C(C)C)[C@H]1CC[C@@H]2[C@@]1(CC[C@H]3C2=C[C@H]([C@@]4([C@@]3(CC[C@@H](C4)O)C)O)O)C
Methyl Lucidenate F	21633085	32.67	0.81	C_28_H_38_O_6_	C[C@H](CCC(=O)OC)[C@H]1CC(=O)[C@@]2([C@@]1(CC(=O)C3=C2C(=O)C[C@@H]4[C@@]3(CCC(=O)C4(C)C)C)C)C
Ganoderic acid beta	10097521	30.54	0.82	C_30_H_44_O_6_	C[C@H](CC/C=C(\C)/C(=O)O)[C@H]1CC(=O)[C@@]2([C@@]1(CC(=O)C3=C2[C@H](C[C@@H]4[C@@]3(CC[C@@H](C4(C)C)O)C)O)C)C
lucidenic acid A	14109375	30.34	0.79	C_27_H_38_O_6_	C[C@H](CCC(=O)O)[C@H]1CC(=O)[C@@]2([C@@]1(CC(=O)C3=C2[C@H](C[C@@H]4[C@@]3(CCC(=O)C4(C)C)C)O)C)C
Methyl lucidenate Q	11271456	30.19	0.81	C_28_H_42_O_6_	C[C@H](CCC(=O)OC)[C@H]1C[C@@H]([C@@]2([C@@]1(CC(=O)C3=C2[C@H](C[C@@H]4[C@@]3(CCC(=O)C4(C)C)C)O)C)C)O

## Data Availability

The data presented in this study are openly available in GEO at https://www.ncbi.nlm.nih.gov/geo/query/acc.cgi?acc=GSE312260 (accessed on 7 July 2026), reference number GSE312260.

## Supplementary Materials

The following supporting information can be downloaded at: https://www.mdpi.com/article/10.3390/ijms27146120/s1.

## Abbreviations

AUC, area under the curve; AURKA, Aurora kinase A; BP, biological process; CC, cellular component; CDK2, cyclin-dependent kinase 2; CRC, colorectal cancer; DEGs, differentially expressed genes; DL, drug-likeness; GEO, Gene Expression Omnibus; GI, gastrointestinal; GO, Gene Ontology; GSVA, gene set variation analysis; KEGG, Kyoto Encyclopedia of Genes and Genomes; KIF11, kinesin family member 11; MD, molecular dynamics; MF, molecular function; MM-PBSA, molecular mechanics–Poisson–Boltzmann surface area; MSigDB, Molecular Signatures Database; NPT, number of particles, pressure, temperature; NVT, number of particles, volume, temperature; OB, oral bioavailability; OMIM, Online Mendelian Inheritance in Man; PDOs, patient-derived organoids; PPI, protein-protein interaction; Rg, radius of gyration; RMSD, root-mean-square deviation; RMSF, root-mean-square fluctuation; ROC, receiver operating characteristic; SASA, solvent-accessible surface area; scRNA-seq, single-cell RNA-sequencing; TCMSP, Traditional Chinese Medicine Systems Pharmacology Database and Analysis Platform; TCGA, The Cancer Genome Atlas Program; UMAP, uniform manifold approximation and Projection; WGCNA, weighted gene co-expression network analysis.
