# Supplementary material for: Targeting CDK2 and AURKA with Cerevisterol from Ganoderma lucidum to Sensitize Colorectal Cancer to Chemotherapy
Source: Int J Mol Sci. 2026 Jul 8;27(14):6120. doi: 10.3390/ijms27146120 (PMC13411116; doi:10.3390/ijms27146120)
Supplement: Supplementary file 1 [file ijms-27-06120-s001.zip › Supplementary materials.pdf]

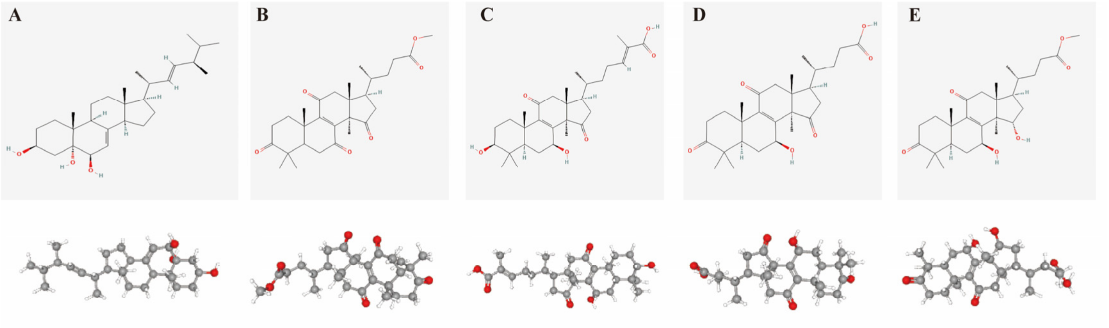

Supplementary Figure S1. Two-dimensional (2D) and three-dimensional (3D) chemical structures of the five bioactive components identified from Ganoderma lucidum. (A) Cerevisterol. (B) Methyl Lucidenate F. (C) Ganoderic acid beta. (D) Lucidenic acid A. (E) Methyl lucidenate Q.

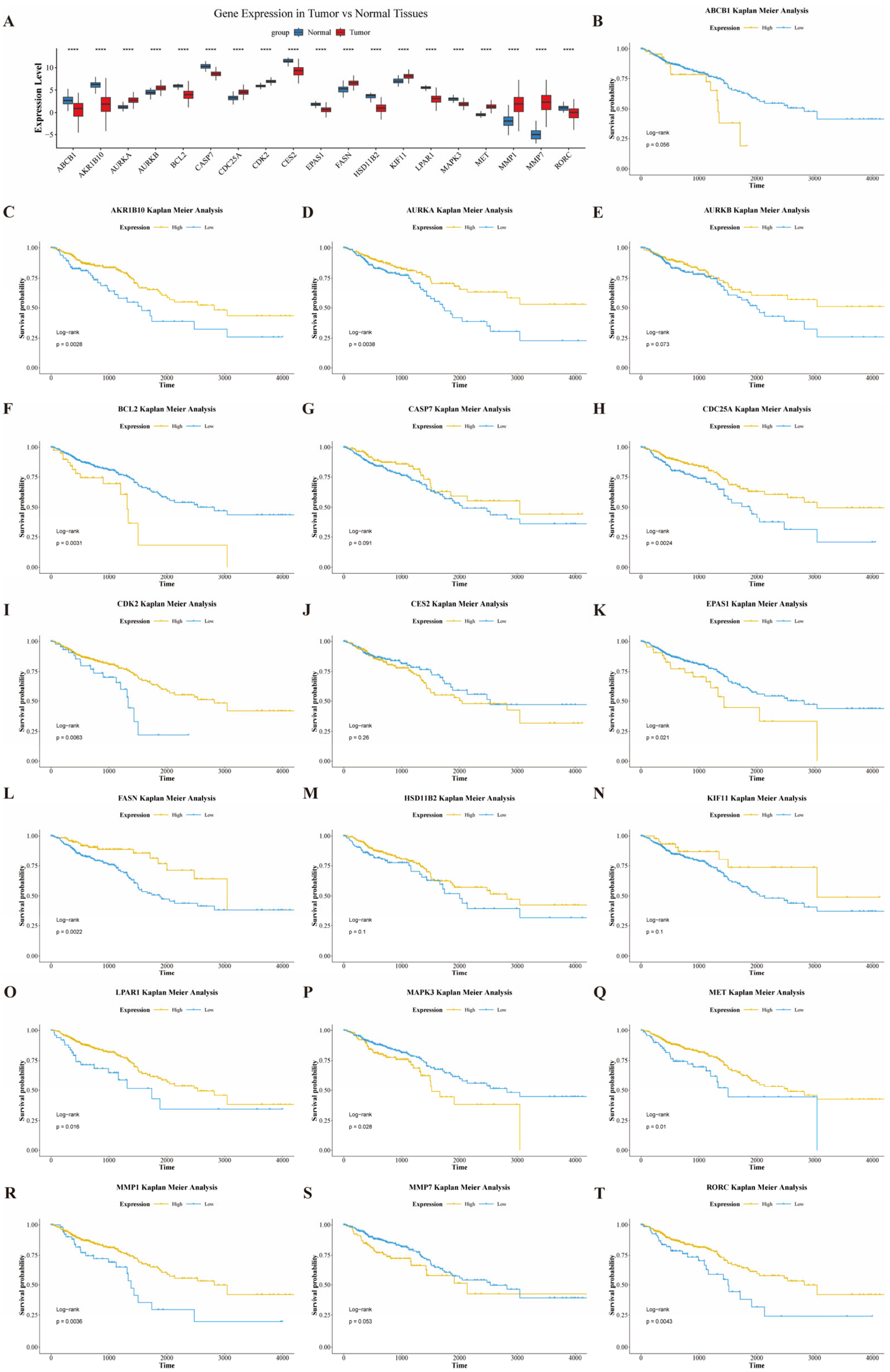

Supplementary Figure S2. Differential expression and survival analysis of the 19 target genes in normal tissues and colorectal cancer patients (*P<0.05, **P<0.01, ***P<0.001, ****P<0.0001).

A. Boxplot showing the differential expression of the 19 genes between tumor and normal samples in the TCGA cohort.

(B–T) Kaplan–Meier survival curves illustrating the association between the expression level of each individual gene (B to T) and patient overall survival.

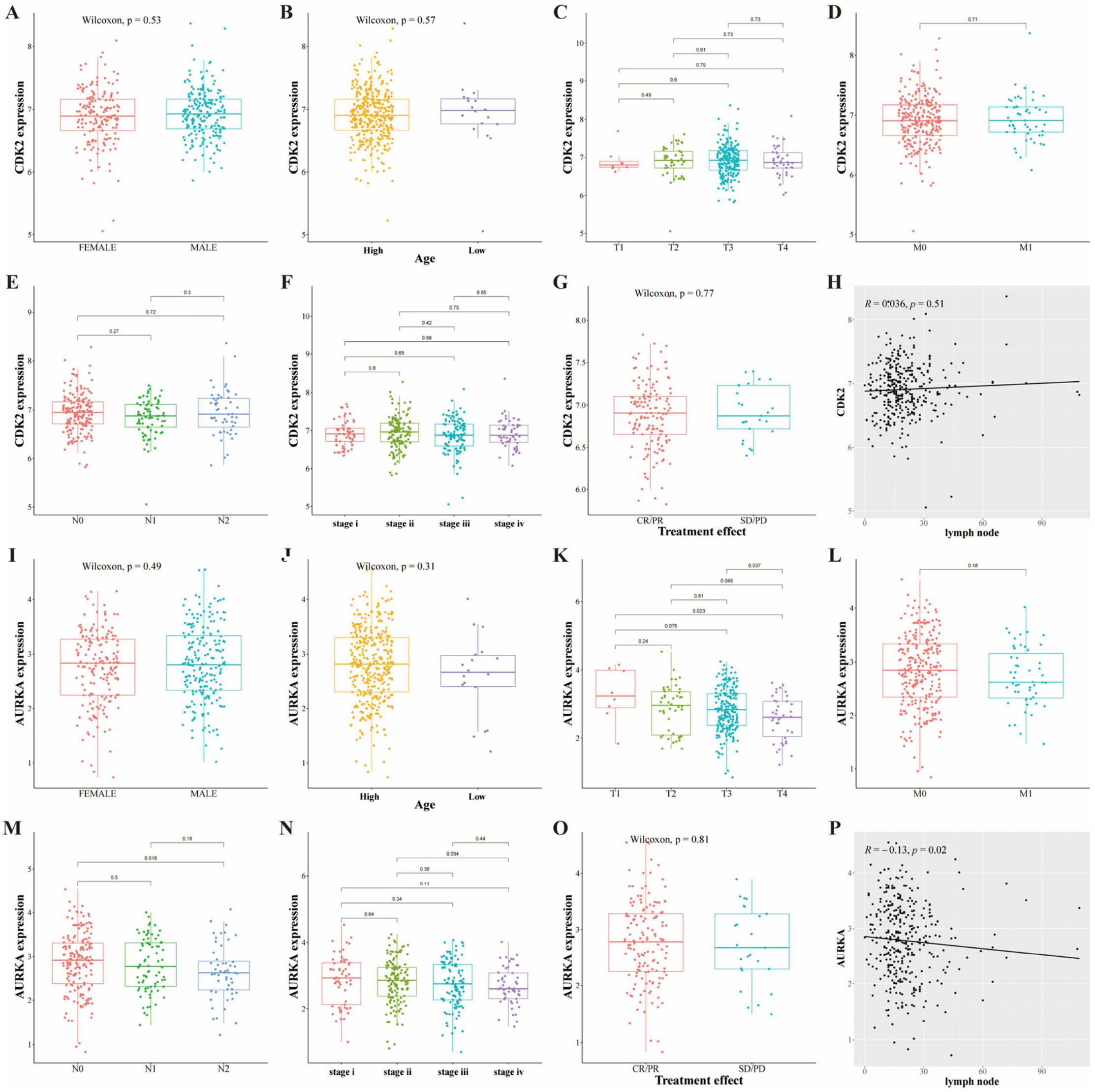

Supplementary Figure S3. Correlation analysis of CDK2 and AURKA expression with clinical characteristics in colorectal cancer.

(A–H) Correlation of CDK2 expression with (A) gender, (B) age, (C) T stage, (D) M stage, (E) N stage, (F) stage, (G) clinical benefit rate, and (H) lymph node count.

(I–P) Correlation of AURKA expression with (I) sex, (J) age, (K) T stage, (L) M stage, (M) N stage, (N) stage, (O) clinical benefit rate, and (P) lymph node count.
